# Imaging the Double Bubble: A Case of Duodenal Atresia Detected in Late Pregnancy

**DOI:** 10.7759/cureus.68759

**Published:** 2024-09-05

**Authors:** Nikita Bora, Pratapsingh Parihar, Nishant Raj, Neha D Shetty

**Affiliations:** 1 Radiodiagnosis, Jawaharlal Nehru Medical College, Datta Meghe Institute of Higher Education and Research, Wardha, IND

**Keywords:** b mode usg, double-bubble, duodenal atresia, pediatric surgery, pediatric ultrasound

## Abstract

Duodenal atresia is a rare congenital gastrointestinal obstruction, usually recognized by a prominent "double bubble" sign on prenatal imaging. This case report presents a diagnosis of duodenal atresia in a fetus in the third trimester. The mother presented late for an antenatal ultrasound, which revealed the classic "double bubble" sign. Postpartum abdominal radiographs confirmed the diagnosis, showing an air-filled, dilated abdomen and proximal duodenum with no distal bowel without any gas. A successful surgical operation was performed. This case highlights the importance of imaging in the diagnosis and timeliness of management of duodenal atresia.

## Introduction

Duodenal atresia is the second most common form of gastrointestinal atresia, occurring in approximately one in 10,000 live births. This condition arises due to a failure in the canalization of the embryonic duodenum, which may be caused by an ischemic event or genetic factors. Unlike other types of intestinal atresia, duodenal atresia is frequently associated with additional congenital anomalies. Notably, around 25% to 40% of infants with duodenal atresia also have Down syndrome. Other associated anomalies may include VACTERL syndrome (vertebral, anal, cardiac, tracheoesophageal, renal, and limb anomalies), malrotation, annular pancreas, biliary tract abnormalities, and mandibulofacial anomalies [1].

Diagnosis of duodenal atresia is often suspected prenatally when polyhydramnios or a dilated stomach is observed. Prenatal ultrasound can reveal a "double bubble" sign, characterized by a large gastric bubble and a smaller proximal duodenal bubble, in up to 80% of cases. Postnatally, affected infants typically present with feeding difficulties and bilious vomiting. The classic "double bubble" appearance on X-ray, with one bubble in the stomach and another in the proximal duodenum and little to no air in the distal gut, further supports the diagnosis. While an upper gastrointestinal series can confirm the diagnosis, it is often unnecessary if surgery is planned immediately. In cases where surgery must be delayed, a contrast enema may be performed to rule out malrotation.

Management involves placing the infant on nothing-by-mouth status, inserting a nasogastric tube to decompress the stomach, and ultimately performing surgery, which is the definitive treatment for duodenal atresia [2].

## Case presentation

Antenatal history

**Figure 1:**
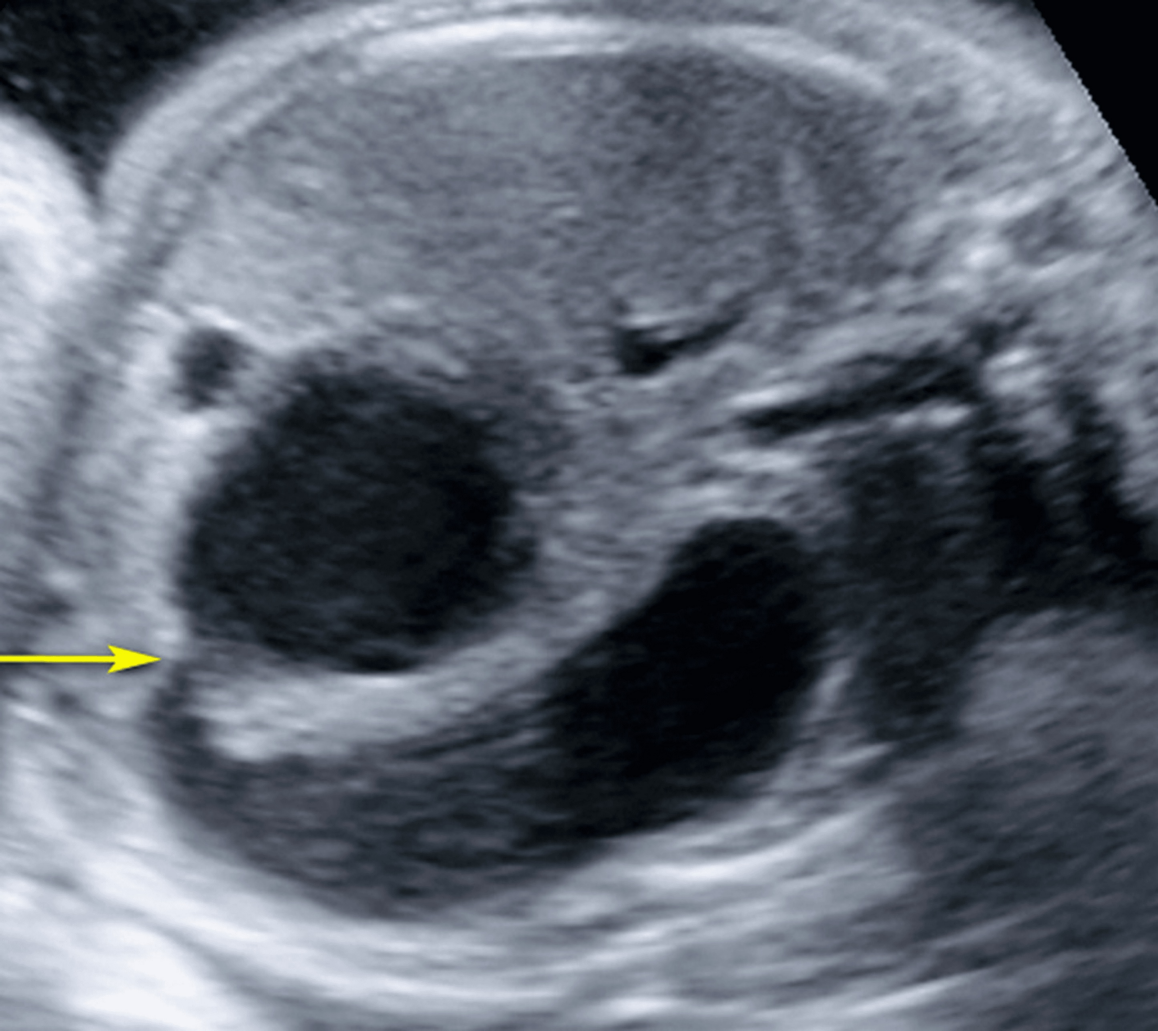
Greyscale ultrasound image showing double bubble sign (yellow arrow).

**Figure 2:**
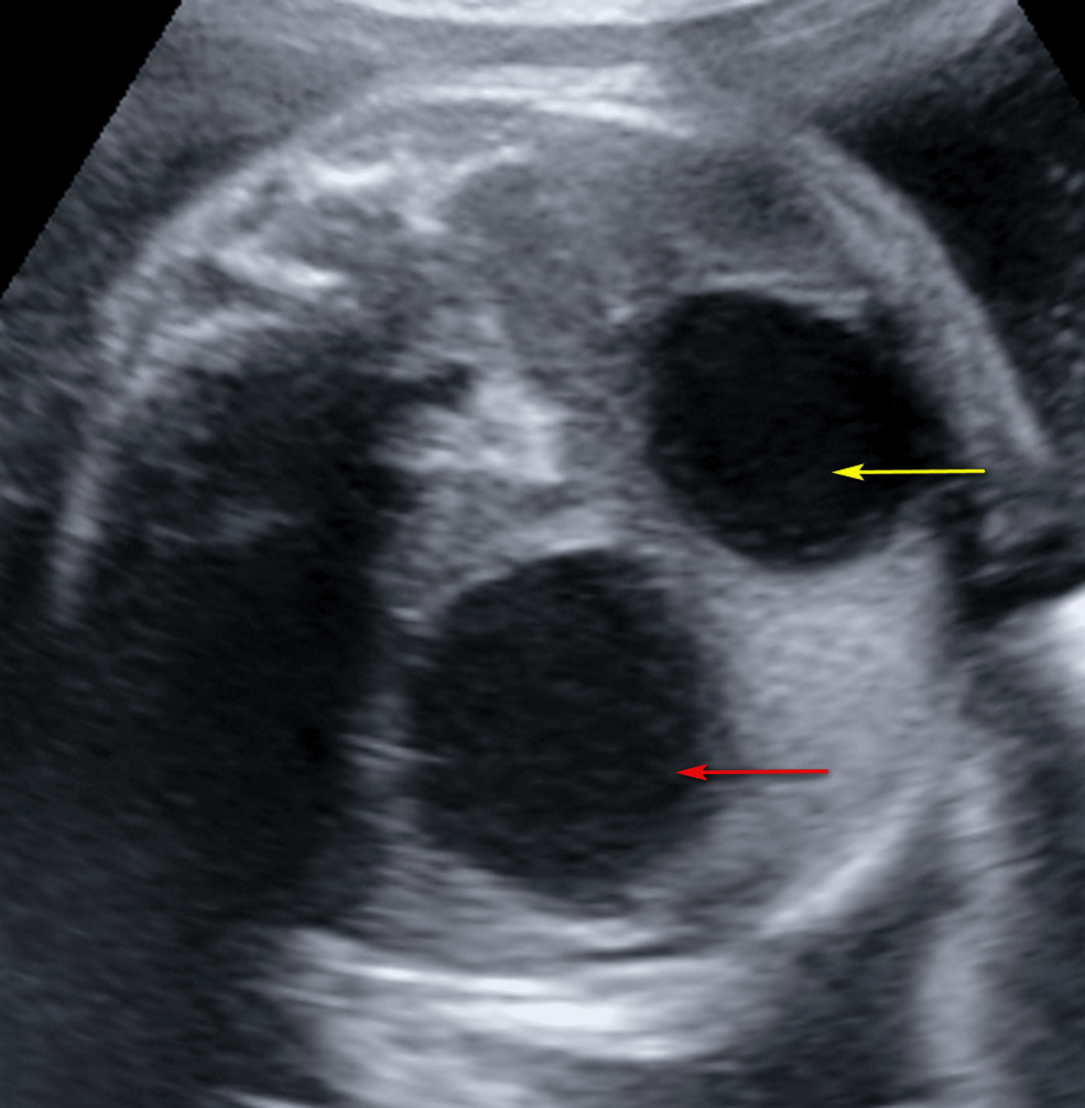
Greyscale ultrasound image showing dilated stomach (yellow arrow) and duodenum (red arrow) giving rise to double sign.

**Figure 3:**
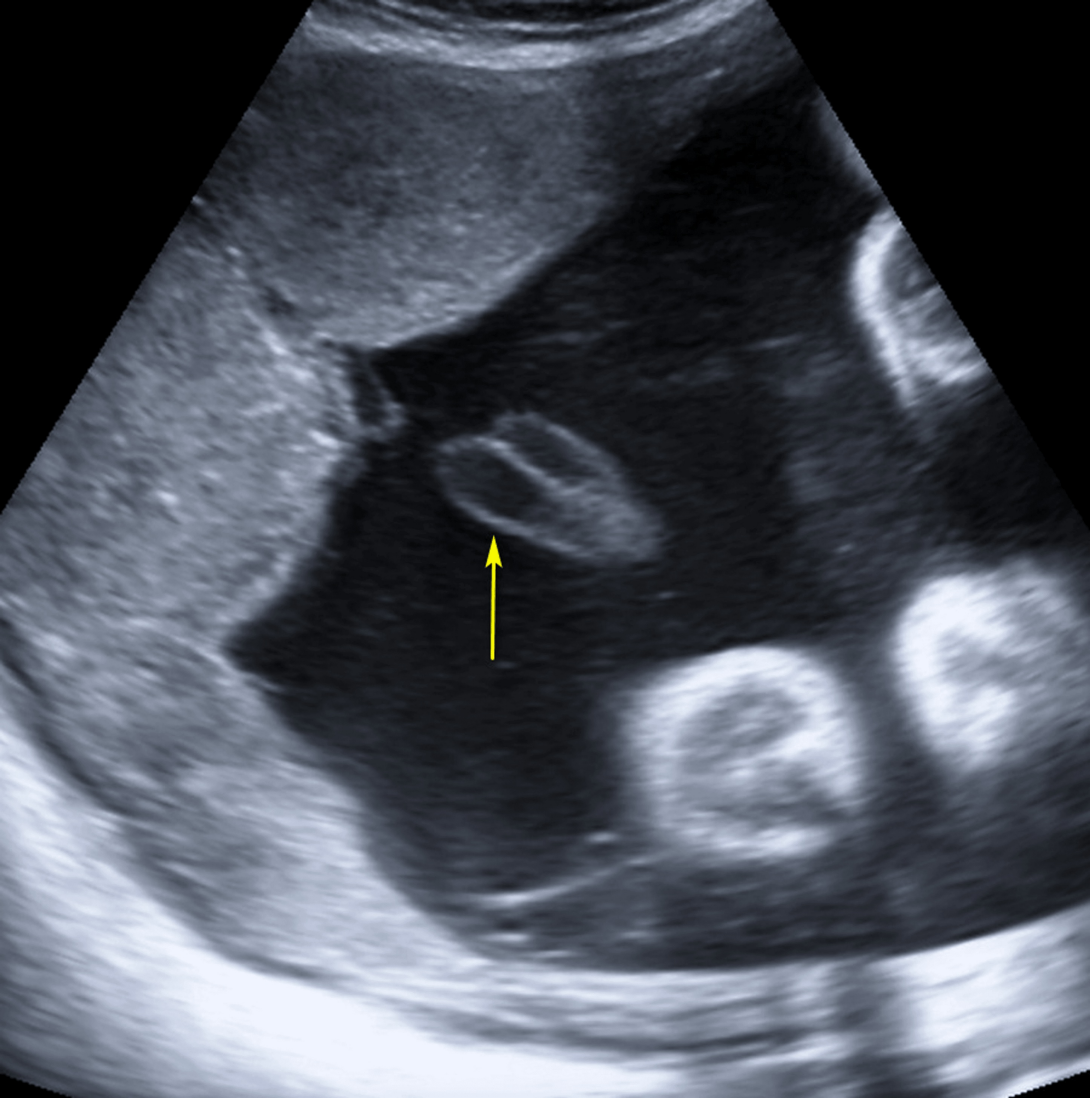
Greyscale ultrasound image showing double vessel sign (yellow arow) indicating presence of only one umbilical artery.

**Figure 4:**
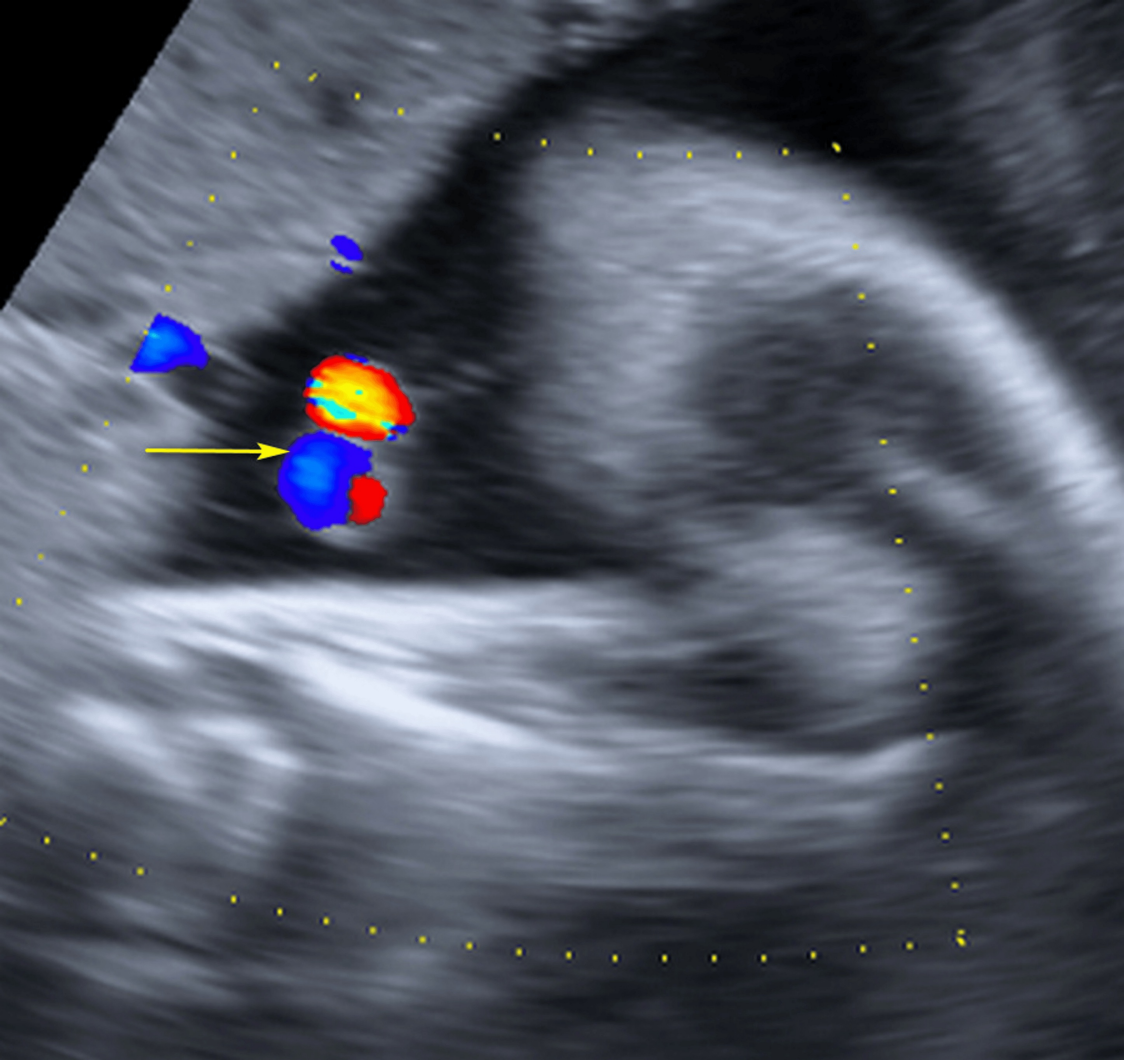
Color Doppler ultrasound image showing double vessel sign (yellow arrow) indicating presence of only one umbilical artery.

The mother first presented to our facility in the late third trimester for an antenatal scan. There were no previous antenatal or anomaly scans conducted. During the antenatal scan, there was a presence of dilated stomach and duodenum giving a double bubble-type appearance (Figures 1, 2) with polyhydramnios. In addition, there was a presence of a double vessel sign in the umbilical cord indicating the presence of only one umbilical artery and vein (Figures 3, 4).

Neonatal details

A 2.36 kg female child was born at 36+6 weeks of gestational age on December 16, 2023, at 11:20 AM via lower segment cesarean section (LSCS). The indication for LSCS included a previous LSCS and the antenatal ultrasound findings of duodenal atresia with polyhydramnios and moderate anemia. The baby cried immediately after birth and was noted to have a congenital anomaly, specifically a tag (extra thumb). She was subsequently shifted to the neonatal intensive care unit (NICU).

**Figure 5:**
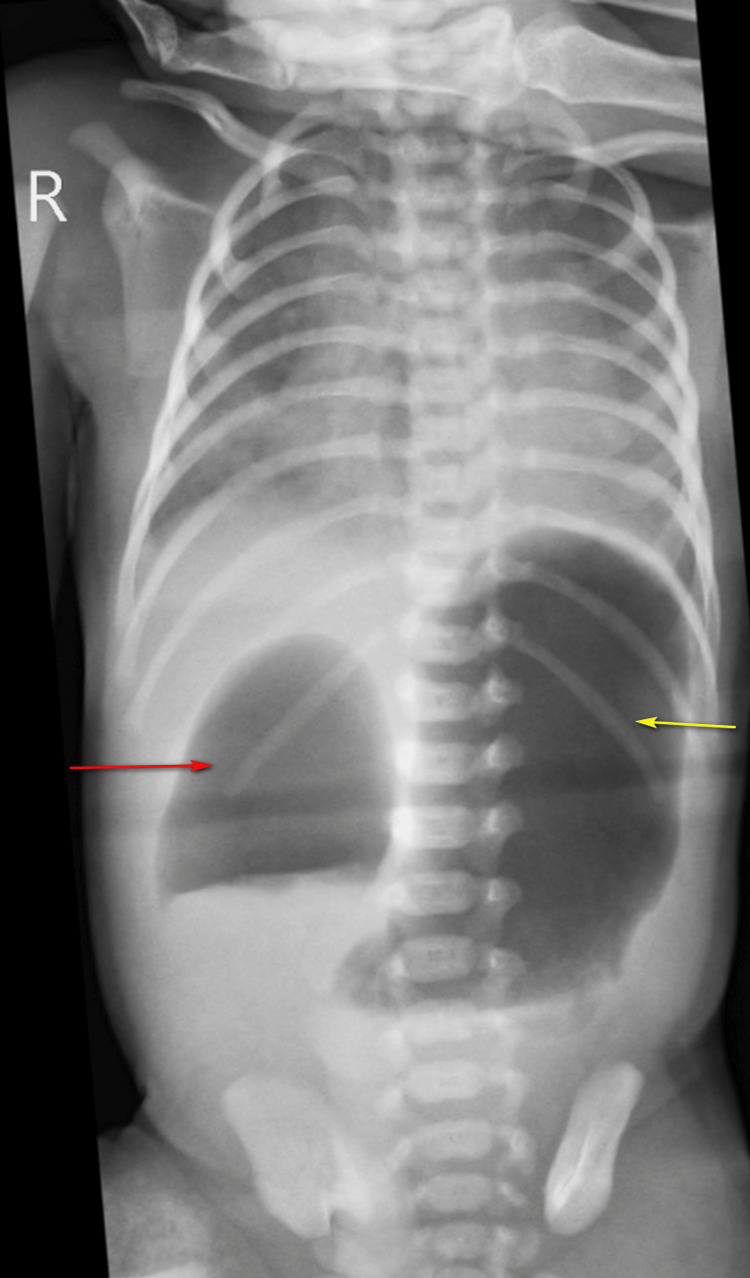
Abdominal radiograph showing air-filled dilated stomach (yellow arrow) and duodenum (red arrow) with absence of distal bowel gas.

Abdominal radiographs revealed a double bubble sign, characterized by a gas-filled, distended stomach and duodenum, with no gas present in the distal intestines (Figure 5).

Neonatal history

Upon admission to the NICU, the baby was kept nil by mouth and started on intravenous (IV) fluids, Inj. Ampicillin, and Inj. Gentamycin. The initial septic screen revealed a hemoglobin (Hb) level of 18.1 g/dL, a total leukocyte count (TLC) of 22,000/mm³, platelets at 1.7 lakh/mm³, C-reactive protein (CRP) at 0.2 mg/L, and kidney function tests (KFT) showed urea of 18 mg/dL, creatinine of 1.2 mg/dL, sodium (Na) of 138 mEq/L, and potassium (K) of 4 mEq/L. The coagulation profile was within normal range, and a blood culture was sent. An abdominal ultrasound confirmed the diagnosis of duodenal atresia.

Surgical intervention

The baby underwent an emergency exploratory laparotomy with duodeno-duodenostomy. Two feeding tubes were placed: one at the surgical anastomosis site and the other in the stomach. The baby was intubated intraoperatively and remained intubated postoperatively. The procedure was uneventful with a minimal blood loss of 12 mL, and 1 unit of packed red cells (PRC) was transfused. Postoperatively, the antibiotics were escalated to Inj. Meropenem, Inj. Colistin, and Inj. Fluconazole and an intralipid infusion were started. Postoperative investigations showed Hb of 18.4 g/dL, TLC of 11,700/mm³, platelets of 1.81 lakh/mm³, urea of 24 mg/dL, creatinine of 1.6 mg/dL, Na of 137 mEq/L, and K of 4.8 mEq/L.

Postoperative course

In the postoperative period, the baby was started on fentanyl infusion, dobutamine infusion, and Vitamin K. A neurological ultrasound (NUSG) was performed, which was normal. A 2D echocardiography revealed a small patent foramen ovale (PFO) with a left-to-right shunt and no evidence of coarctation of the aorta. A repeat KFT showed an increase in creatinine to 2.5 mg/dL, necessitating adjustment of the Colistin dose based on renal clearance. The baby exhibited altered gastric aspirates, and the complete blood count (CBC) showed Hb of 12.7 g/dL, TLC of 10,100/mm³, and platelets of 0.30 lakh/mm³. Fresh frozen plasma (FFP), PRC, and platelets were transfused, and antibiotics were upgraded to Inj. Amphotericin B. The baby experienced episodes of desaturation and endotracheal bleeding, necessitating reintubation and administration of multiple platelets. Minimal orogastric (OG) feeds were initiated, and the baby was extubated, initially receiving oxygen via high-flow nasal cannula (HFNC), then transitioning to oxygen via hood to nasal prongs. The baby had difficulty tolerating feeds and continued to exhibit daily evidence of gastric aspirates. A repeat CBC showed Hb of 13.8 g/dL, platelets of 0.35 lakh/mm³, CRP of 44 mg/L, creatinine of 1.3 mg/dL, and INR of 1.11. Additional medications including Tab Jr Lanzol, Motinorm drops, and Inj. MgSO₄ was administered.

**Figure 6:**
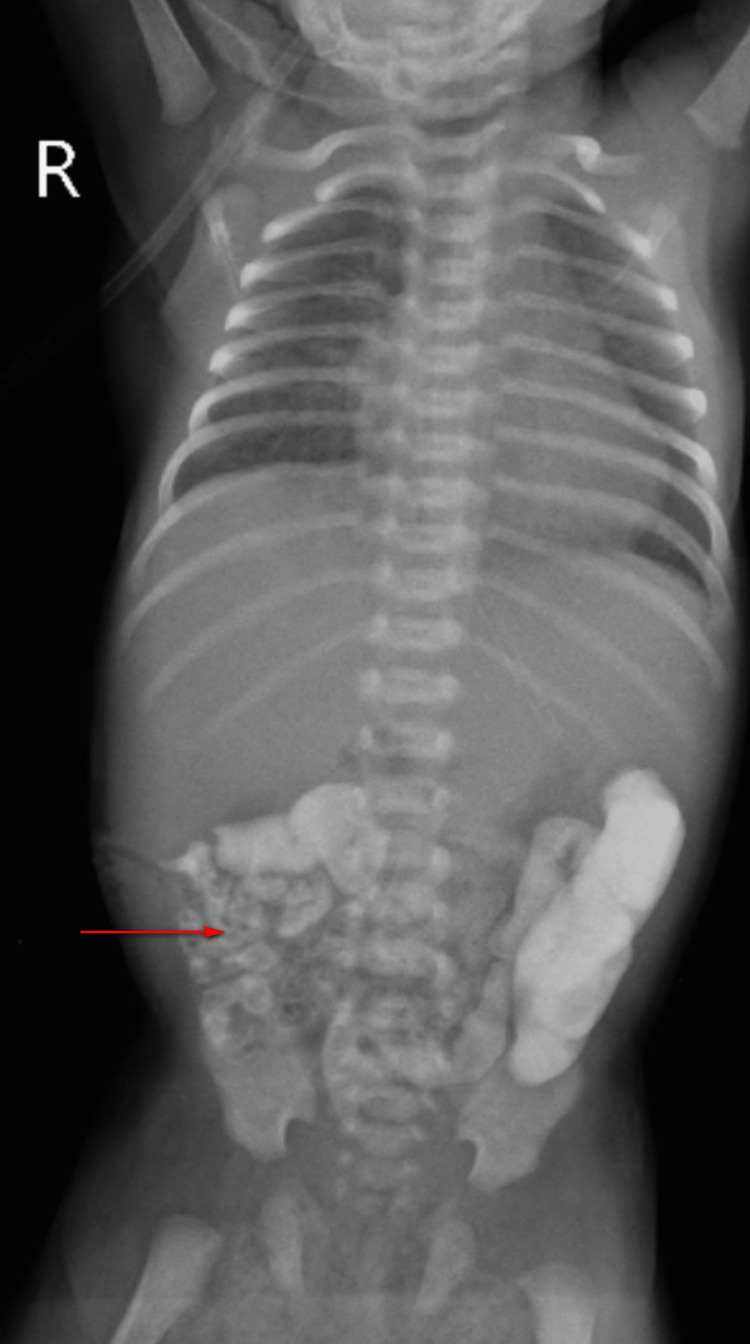
Postoperative dye study showing presence of contrast in distal bowel loops (red arrow) suggesting patency.

A dye study (Figure 6) of the abdomen confirmed patent intestinal motility and Tab Erythromycin was added to enhance intestinal motility. The second blood culture showed no growth.

Complications and subsequent management

The baby’s extra thumb (tag) became necrotic and was excised by the pediatric surgeon. The third blood culture revealed Methicillin-Sensitive *Staphylococcus aureus* (MSSA). Subsequently, the baby developed tachypnea and respiratory distress, along with an abscess over the left arm, which required oxygen via CPAP. Antibiotics were upgraded to Inj. Vancomycin and Inj. Tigecycline and Tab Erythromycin were discontinued. Chest physiotherapy was initiated, and a repeat CBC showed Hb of 6.8 g/dL, TLC of 18,300/mm³, and platelets of 0.05 lakh/mm³. PRC and multiple platelets were transfused, and oxygen was gradually tapered to nasal prongs. Incision and drainage of the arm abscess were performed, and pus culture revealed Klebsiella. Vitamin D₃ drops were added, and OG feeds were gradually increased to full feeds, allowing for the tapering and discontinuation of IV fluids. The baby experienced multiple episodes of tachycardia, managed with a Neomol suppository. Vancomycin and Tigecycline were administered for seven days, and Syrup Septran was started with plans to continue for 10 days. Screening for retinopathy of prematurity (ROP) and a follow-up NUSG were both normal. Breastfeeding (BF) and Kangaroo care (KC) were successfully established. The baby later developed swelling on the back of the head (left side), and a pediatric surgeon recommended an ultrasound, which suggested an infective etiology, with a follow-up advised. After completing nine days of Septran, with one more day advised, the baby was found to be vitally and hemodynamically stable.

Discharge

The baby was discharged in a stable condition.

## Discussion

In our case, the mother presented for the first time in the late third trimester, with no previous antenatal or anomaly scans. The antenatal ultrasound revealed the "double bubble" sign, strongly suggesting duodenal atresia. The baby, weighing 2.36 kg, was delivered via LSCS at 36+6 weeks due to the antenatal diagnosis of duodenal atresia and polyhydramnios in the context of a previous LSCS. Postnatally, the baby was admitted to the NICU, where duodenal atresia was confirmed via abdominal ultrasound, and an emergency exploratory laparotomy with duodeno-duodenostomy was performed immediately. The postoperative course included various complications, such as altered gastric aspirates, desaturation episodes, and an arm abscess, which were managed with appropriate surgical and medical interventions. Despite these challenges, the baby was stabilized and discharged in good condition.

In a similar case reported by Arruarana et al., a 28-year-old G5P3013 woman was initially assessed with a normal anatomy scan and non-invasive prenatal testing (NIPT) at 22 weeks of gestation. However, by 31 weeks, the patient presented with significant polyhydramnios, and a sonogram revealed an enlarged stomach. One week later, the classic "double bubble" sign indicative of duodenal atresia was detected. Despite the findings, the patient declined amniocentesis. A fetal echocardiogram showed biventricular hypertrophy, prompting genetic and neonatal consultations. The infant was delivered vaginally at 38 weeks, weighing 2810 g, with APGAR scores of 8/9/9. Postnatally, an X-ray confirmed the diagnosis of duodenal atresia, and the neonate was transferred to another hospital for surgical repair. During surgery, a dilated proximal duodenum, a collapsed distal duodenum, and bowel malrotation were identified. The surgical procedures included a Ladd's procedure and duodeno-duodenostomy [3].

Another case reported by Pasam et al. was of a preterm infant diagnosed with duodenal atresia who presented with non-bilious vomiting on the third day of life. This presentation is unusual, as the majority of duodenal atresia cases manifest with bilious vomiting due to the atresia occurring distal to the ampulla of Vater. In this case, the absence of bilious vomiting indicated that the atresia was located proximally, consistent with a preampullary atresia. Although the infant’s antenatal sonography suggested duodenal atresia, postnatal abdominal radiographs did not show the classic double bubble sign. Instead, an upper gastrointestinal contrast study revealed a dilated stomach and the first part of the duodenum, leading to the diagnosis. During laparotomy, the surgeons noted malrotation of the midgut and a narrowing at the first and second parts of the duodenum without a complete discontinuity of the bowel wall. A Kimura-type duodeno-duodenostomy was successfully performed, and the infant made an uneventful recovery. In contrast, our case involved a full-term infant diagnosed with duodenal atresia late in the third trimester through antenatal ultrasound, which revealed the classic double bubble sign. Unlike the case reported by Pasam et al., our infant did not present with non-bilious vomiting, likely due to the atresia being distal to the ampulla of Vater [4].

Congenital intestinal obstructions are classified as intrinsic (atresia, stenosis, web) or extrinsic (malrotation, Ladd's bands, annular pancreas, etc.). Among these, malrotation is the most immediately life-threatening due to the risk of midgut volvulus.

Duodenal atresia, the most common form of intrinsic obstruction, occurs in about one in 5,000 to 10,000 live births. Duodenal stenosis is less common, and malrotation may also occur alongside intrinsic obstructions. The duodenum forms from the caudal foregut and cranial midgut, merging just distal to the ampulla of Vater. Duodenal atresia results from the failure of the duodenal lumen to recanalize during the 8th to 10th week of gestation. Incomplete recanalization can cause stenosis or a duodenal web.

Most obstructions occur distal to the ampulla of Vater, leading to bilious vomiting. However, about 20% of cases present with non-bilious vomiting due to a proximal obstruction. Duodenal atresia can be diagnosed antenatally in up to 50% of cases, typically identified by polyhydramnios and stomach/proximal duodenum dilation in the third trimester. After birth, complete obstructions usually present within 24 hours, with persistent vomiting. Incomplete obstructions often present later, with more variability, making diagnosis more challenging. These conditions can be mistaken for more common issues like pyloric stenosis or gastroesophageal reflux due to recurrent vomiting [5].

## Conclusions

In summary, this case of duodenal atresia was identified late in the third trimester through an antenatal scan showing the classic "double bubble" sign. The neonate, born via cesarean section, underwent a successful duodeno-duodenostomy and experienced a complex postoperative course, including multiple infections and complications. Despite these challenges, the infant was eventually stabilized and discharged, highlighting the importance of timely surgical intervention and vigilant postoperative care in managing this congenital condition.
